# Autism Spectrum Disorder in Children with an Early History of Paediatric Acquired Brain Injury

**DOI:** 10.3390/jcm12134361

**Published:** 2023-06-28

**Authors:** Melanie Porter, Sindella Sugden-Lingard, Ruth Brunsdon, Suzanne Benson

**Affiliations:** 1 School of Psychology, Faculty of Medicine, Health and Human Sciences, Macquarie University, Sydney, NSW 2109, Australia; 2 Kids Rehab, The Children’s Hospital at Westmead, SCHN, Westmead, NSW 2145, Australia

**Keywords:** autism, paediatric, acquired brain injury, risk factor

## Abstract

Autism spectrum disorder (ASD) is a neurodevelopmental condition that arises from a combination of both genetic and environmental risk factors. There is a lack of research investigating whether early acquired brain injury (ABI) may be a risk factor for ASD. The current study comprehensively reviewed all hospital records at The Brain Injury Service, Kids Rehab at the Children’s Hospital at Westmead (Australia) from January 2000 to January 2020. Of the approximately 528 cases, 14 children with paediatric ABI were subsequently given an ASD diagnosis (2.7%). For this ASD sample, the mean age at the time of the ABI was 1.55 years, indicating a high prevalence of early ABI in this diagnostic group. The mean age of ASD diagnosis was, on average, 5 years later than the average ASD diagnosis in the general population. Furthermore, 100% of children had at least one medical comorbidity and 73% had three or more co-occurring DSM-5 diagnoses. Although based on a small data set, results highlight early paediatric ABI as a potential risk factor for ASD and the potential for a delayed ASD diagnosis following early ABI, with comorbidities possibly masking symptoms. This study was limited by its exploratory case series design and small sample size. Nonetheless, this study highlights the need for longitudinal investigation into the efficacy of early screening for ASD symptomatology in children who have sustained an early ABI to maximise potential intervention.

Autism spectrum disorder (ASD) is a complex neurodevelopmental condition that arises from a combination of genetic and environmental risk factors [1,2]. Despite extensive research into the mechanisms responsible for ASD development, the exact patho-etiological mechanisms remain unknown. ASD is thought to be polygenetic and heterogeneous, with several genes being implicated, including *NLGN* and *SLC6A4* [3], and replicability being found for several chromosomal loci including 2q, 5, 7q, 15q, and 16p [4]. Several early environmental risk factors for ASD have been implicated, including maternal gestational diabetes [5], elevated maternal prenatal stress [6], advanced maternal and paternal age, [7], and birth complications, such as those resulting in hypoxic-ischemic encephalopathy [8]. Despite the well-established literature linking perinatal hypoxic-ischemic encephalopathy and later development of ASD, there has been limited research into whether early acquired brain injury (ABI), more generally, is an environmental risk factor for ASD, with only one study looking at this to date [9], despite suggestions of shared neurobiological mechanisms [10].

Research suggests that predisposing neurobiological factors, such as disruptions to brain structures involved in social information processing and social motivation, and other regions implicated in ASD, such as the cerebellum, may increase the likelihood of an ASD diagnosis [10,11]. Indeed, along with neurodevelopmental conditions such as attention deficit hyperactivity disorder (ADHD) and Down syndrome (DS), which have high comorbidity with ASD, the brain areas involved in social information processing are more susceptible to being damaged during ABI [7,12,13]. Thus, early ABI, impacting on the normal development of specific brain regions involved in social cognition, may place children at a greater risk for later developing ASD.

At this stage, research on whether early ABI poses a risk for the later development of ASD is extremely limited. Chang et al. [9]’s study appears to be the only study that has addressed this topic to date and focused on traumatic brain injury (TBI). Chang et al. conducted a longitudinal study involving a ten-year follow up of a sample of 7801 children who sustained a TBI before the age of three years in Taiwan and who had a subsequent diagnosis of ASD, ADHD, or developmental delay (DD) by board-certified psychiatrists (based on clinical judgment and diagnostic interview). The authors found the incidence of ASD was 0.8% (similar to the internationally reported prevalence rate of 0.6 to 1%; [14], and significantly higher than a control group without TBI (*n* = 31,204 with a rate of 0.4%). Although further research is needed, and despite some methodological shortcomings, Chang et al. suggested that early TBI is a potential risk factor for ASD.

In light of the above, the present study aimed to examine the neurological, developmental, and neuropsychological trajectory of individuals with a history of paediatric ABI who were subsequently diagnosed with ASD. The goals were to report the prevalence of ASD in the paediatric ABI sample, to determine whether the age at which the child sustained an ABI was important, and to establish whether the age of ASD diagnosis was similar to those with ASD and no history of paediatric ABI. Based on the limited research available in the general population on co-occurring medical and mental health conditions, consistent with Levy et al. [15] and Stevens et al. [16], it was hypothesised that the average age of ASD diagnosis would be later in the examined children compared to the average age of diagnosis in the literature due to masking effects.

## 1. Introduction

### 1.1. Diagnostic Criteria for ASD

ASD is a neurodevelopmental condition that involves deficits in social and communication skills and restrictive and repetitive behaviours (DSM-5; American Psychiatric Association, 2013). ASD prevalence rates vary, with the worldwide prevalence estimated to be somewhere between 0.60% and 1.85 % [14].

When following practice standards as outlined in Whitehouse et al., 2018, ASD symptomology can be observed before the age of three years, and ASD can be diagnosed as early as 18 months of age [17]. In a recent review, Van’t Hof et al. [18] reported that across nine studies (*n*  =  18,134), the mean age of ASD diagnosis for children below 10 years of age was 43.18 months (range: 30.90–74.70 months) and in studies that included individuals up to the age of 20 years (35 studies M =  66,966), the mean age of diagnosis was 60.48 months (range: 30.90–234.57 months). Children who have ASD and comorbid diagnoses (e.g., ADHD or ODD) were typically diagnosed with ASD significantly later than those with ASD alone [15,16]. One study found that children who had ADHD and ASD were diagnosed, on average, three years later than those with ASD alone and that children with primary ADHD were 30 times more likely to receive their ASD diagnosis after the age of six years [19].

### 1.2. Aetiology of ASD

#### 1.2.1. Genetics of ASD

Over 100 genes and genomic regions are associated with ASD, largely based on the study of heterozygous, germline, and de novo mutations, as well as copy number variations [14,20,21]. The majority of prenatally expressed genes that increase the risk of ASD are either broadly expressed regulatory genes that occur in the brain and other organs or genes that only occur in the brain [22]. ASD-associated risk genes disrupt cortical wiring, including neuronal outgrowth, synaptogenesis, and neural networks organisation, particularly in the third trimester to early post-natal life [22].

#### 1.2.2. Heritability of ASD

In a meta-analysis, Tick et al. [23] found the heritability correlations for monozygotic twins was 0.98, while the dizygotic correlation was 0.67. Using ascertainment corrections and maximum likelihood estimations, Tick et al. similarly found that the overall heritability ranged from 64% to 91%. Despite a strong biological link to ASD, research also suggests that 40% to 50% of the variance in ASD is determined by environmental factors [24,25], which may include ABI [10].

### 1.3. Models of ASD

Different theories and models attempt to explain the behavioural and developmental manifestations of ASD, but many lack specificity for ASD or a developmental focus. Two of the few models that at least partially overcome these limitations are Singletary’s [2] model and Dennis et al.’s [26] model. Singletary’s model, the “Emotional and Allostatic Overload Model of ASD”, incorporates both genetic and environmental factors and incorporates both pre- and post-natal factors into the development of ASD. This model proposes that allostatic overload (cumulative burden of chronic stress and life events) plays a key role in the development of ASD by amplifying neurobiological vulnerabilities that make contributions to ASD. Dennis et al.’s “Biopsychosocial model” uses biological factors (e.g., prenatal disruption to brain development), environmental factors (e.g., neglect, trauma, abuse, chronic illness, and brain insults), and cognitive and socio-emotional functions to explain child development.

#### Pre-Natal and Postnatal Factors

Neurobiological dysfunction can lead to experiences of environmental deprivation. These dysfunctions can result from a variety of environmental and biological pre-natal risk factors, including gestational diabetes mellitus, neonatal hypoxia, less than the 12-month interpregnancy interval, maternal age of above 40, valproate use during pregnancy, pre-term birth, maternal obesity, elevated levels of foetal cortisol and maternal stress, birth asphyxia, and a maternal history of childhood abuse [8,14,27,28,29,30].

Predisposing neurological factors can impact the brain’s structural development, including regions involved in social information processing and social motivation [31]. These regions are particularly vulnerable during early infancy due to the rapid development of these regions in the prenatal stage [31].

The neurobiology of ASD is still an emerging field. However, negative impacts on the brain’s structures involved in social information processing and social motivation are proposed to lead to lower social engagement and attention to others, which can further hinder the development of an already under-developed social and linguistic brain circuit [10,31]. The brain structures implicated in the social brain network [32] involve the frontal temporal-limbic circuit [31]. Due to the rapid development of these regions in prenatal and early postnatal child development and their importance for social attachment, they are particularly vulnerable to disruptions in the case of paediatric ABI (e.g., paediatric stroke, traumatic brain injury, neurological illness/infection) [10,31]. The cerebellum has also been implicated in social processing and communication and behaviour, all of which are core features of ASD [14]. The cerebellum is also commonly impacted in individuals with TBI [7,12] and ABI [10], and injury to the cerebellum in preterm infants has also been found to be associated with an increased risk of ASD [33].

This early exposure and/or deprivation, in turn, can lead to elevated levels of early life stress (both psychological and allostatic overload); and experience-dependent synapses, changing the wiring of the brain [34]. The interaction between environmental deprivation and psychological stress and allostatic overload, in addition to predisposing biological factors, results in maladaptive neuroplasticity.

### 1.4. ABI as a Risk Factor

Perhaps surprisingly given the above, few studies have examined whether paediatric ABI is an early risk factor for ASD. Chang et al. [9] conducted a ten-year longitudinal study that examined over 31,204 children with no TBI (controls) and 7801 children with TBI before the age of 3 years who had no history of ADHD, ASD, or DD before enrolment in the study. ADHD, ASD, and DD were identified by board-certified psychiatrists based on their clinical judgment and diagnostic interview during the follow-ups. The authors found that after adjusting for demographic and peri-natal conditions, the risk of ASD, along with ADHD and DD, was significantly higher in those with a pre-existing TBI that occurred prior to three years of age compared to controls. Further, children who had a TBI before the age of one year had a higher risk of subsequent ADHD, ASD, or DD than children who experienced a TBI later (between 1 and 3 years of age).

Findings from this study need to be considered alongside several strengths, but also a number of study limitations. Strengths included the large sample size, the longitudinal design, high rates of long-term follow-up (99%), and novelty of the study. Limitations included subjective classification of TBI severity not in line with clinical guidelines, a lack of information on TBI mechanisms and the early traits of neurodevelopmental disorders, and a vague description of the mechanism of ASD diagnosis; also, the rate of ASD in the control group was relatively low. In addition, TBI severity using the Glasgow Coma Scale (GCS) was not obtained and, thus, the authors defined severe TBI as cases that required “neurosurgical intervention” and mild TBI as cases that “did not receive neurosurgical intervention and were not admitted to the hospital” for their TBI. This method of classification is a limitation of this study, as not using an evidence-based severity measure is against clinical guidelines [35], and further, not using a conventional severity rating could lead to a mislabelling of TBI severity and thus inaccurate interpretation of results. There is also no mention of how any moderate severity classifications of TBI were given, but moderate classifications were used in the study’s demographic descriptions and results. Chang et al. [9] found that risks of ADHD, ASD, and DD increased after severe TBI compared with mild and moderate TBI; however, this result is difficult to interpret due to the criteria used to classify TBI severity.

This study also found that the risks of ADHD, ASD, and DD were significantly higher after repeated TBI events, but they did not specify how many children had more than one TBI event. It would be uncommon for children to have multiple TBI events before the age of three years (Sariaslan et al. [36] found that 12.5% of individuals had recurrent TBI’s before the age of 25 years), and the number of children in this sub-category is likely small. Data were not collected on other possible medical comorbidities or the presence of other neurodevelopmental conditions due to not having access to the full medical records of participants. Thus, it is unclear if the results may have been impacted by other comorbid medical and/or neurodevelopmental conditions that were not mentioned.

### 1.5. Current Study

Despite theories and models of ASD suggesting early life neurological compromise (e.g., hypoxic injuries at birth) may play a role in increasing the risk of ASD [2,26], there is presently limited research into whether an ABI sustained early on in life is a risk factor for ASD. The present exploratory research contributed to the scientific literature on this topic by comprehensively examining medical records at Kids Rehab (The Children’s Hospital at Westmead, Sydney, Australia) across a 20-year period.

The three aims of the current research were:

1. To report the prevalence of ASD in the paediatric ABI sample.
2. To determine whether the age at which the child sustained an ABI was important.
3. To establish whether the age of ASD diagnosis was similar to those with ASD and no history of paediatric ABI and whether the presence of comorbidities impacted on the age of the ASD diagnosis.

The following was hypothesised in relation to these aims:

1. The prevalence of ASD in a paediatric ABI sample would be higher than in the general population.
2. In line with the vulnerability hypothesis, it was predicted that age of ABI would be an important factor in determining whether ASD was present, with a younger age at ABI leading to a higher prevalence of co-occurring ASD [36].
3. In line with Levy et al. [15] and Stevens et al. [16], it was predicted that the age of diagnosis of ASD would be later than the age of ASD diagnosis in the general population (which is 60.48 months, range: 30.90–234.57 months; Van’t Hof et al. [18], and that medical and/or DSM-5 comorbidities would be an important factor in delayed age of ASD diagnosis [15,16].

## 2. Materials and Methods

### 2.1. Study Design

This present study systematically and comprehensively examined medical records from January 2000 to January 2020 at The Brain Injury Service Kids Rehab at the Children’s Hospital at Westmead (Sydney, Australia) to look for the prevalence of ASD and to identify cases to be chosen for further examination (which was undertaken at the end of the study period—2020/2021).

### 2.2. Procedures

Electronic records from The Brain Injury Service (Kids Rehab) at the Children’s Hospital at Westmead, were searched to identify those children who were admitted with an ABI between January 2000 and January 2020 (approximately 500 cases). The Australian Institute of Health and Welfare definition of ABI was used (AIHW, 2006), in which ABI is defined as “multiple disabilities arising from damage to the brain acquired after birth. It results in deterioration in cognitive, physical, emotional or independent functioning. It can be as a result of accidents, stroke, brain tumours, infection, poisoning, lack of oxygen, degenerative neurological disease, etc.” (AIHW 2006).

Prevalence rates were obtained via examination of the number of active cases at Kids Rehab and the number of children with an ASD diagnosis in addition to an ABI. The keywords “autism” and “ASD” were separately typed into the search function to electronically identify ABI children diagnosed with ASD. Pervasive Developmental Disorder-Not Otherwise Specified (PDD-NOS) as per the DSM-IV (American Psychiatric Association, 2000) was not used as a search criterion due to this diagnosis no longer being relevant for the DSM-5 (American Psychiatric Association, 2013). All documents that included the search terms were then individually screened by the first author to identify appropriately diagnosed ASD cases. Children were included in the present study if they had a confirmed diagnosis of ASD either by a paediatrician or through formal standardised ASD testing (such as the ADOS); all children had a confirmed history of paediatric ABI. Once cases were identified, demographic and medical information was comprehensively reviewed by the first author, along with allied health reports and any psychometric test data. The Sydney Children’s Hospital Network Human Research Ethics Committee approved this research (HREC: 2020/ETH01984).

### 2.3. Participants

The present study tracked retrospective, longitudinal, neuropsychological assessments and medical records of 14 children (*n* = 7 males, *n* = 7 females) with a history of paediatric ABI that occurred between their birth to 9 years and 10 months, and who attracted a subsequent diagnosis of ASD between the ages of 4 and 15 years.

### 2.4. Measures

The following demographic information was extracted from medical records and allied health reports for further analysis: sex; chronological age at the time of ABI occurrence, ASD diagnosis and neuropsychological assessment(s); school grade level at the time of any neuropsychological assessment(s); school type attended; birth history and developmental milestones; psychosocial history; family history; and service access. The following medical information was also extracted: the presence of comorbid diagnoses; co-morbid medical or neurological conditions (aside from ABI and ASD); mental health history; the presence of behavioural issues; medications; brain imaging records; and sleep quality. Information regarding the ABI was also retrospectively analysed, including the nature of the ABI, the age when the ABI was sustained, and any brain imaging findings that were available on medical records. Regarding ASD diagnosis, information including the following was extracted: chronological age at the time of the ASD diagnosis; ASD severity level (as per DSM-5; American Psychiatric Association, 2013); age of onset and nature of first ASD symptoms and ASD symptomology. The above information was collected to provide a comprehensive overview of any comorbid conditions and external factors that could be relevant in identifying common patterns across the cases. Neuropsychological test data were also extracted by the first author for the following standardised measures (refer to individual test manuals for further information on these measures, including their psychometric properties):

General Ability: WISC-IV/V [37,38] and/or WPPSI-IV [39] and DAS-II [40];
Academic Achievement: WIAT-II [41].
Attention: CPT-II [42], Tea-CH [43] and the Conners-3 and Early Childhood [44,45,46];
Memory: CMS [6] and CVLT-II/C [47,48];
Executive functioning: TEA-Ch [43], NEPSY-II [49] and the BRIEF-P/II Parent versions [50]; and
Adaptive Functioning: BASC-2/3 Parent [51,52].

Additionally, the Autism Diagnostic Observation Schedule (ADOS; [14]) was reportedly conducted on 6 children. All neuropsychological test scores were converted to *z*-scores, with *z*-scores above −1.64 being considered intact and *z*-scores at or below −1.64 (5th percentile) being considered impaired [53,54]. For the BRIEF-P/II Parent, CPT-II, BASC-2/3 Parent and Conners 3/EC, scores below 1.64 were considered intact and scores at or above 1.64 were considered impaired due to the reversed scoring of these measures [53,54].

## 3. Results

Medical and demographic information is presented in Table 1. Table 2 also provides neuropsychological test results by domain for the most recently available neuropsychological assessment for each child. For a detailed write up of each case, please see the Appendix A (Table A1) and Appendix B.

### 3.1. Summary of Cases

#### 3.1.1. Prevalence Rates

In 2020 and 2021, Kids Rehab had 532 and 523 active ABI patients, respectively. With an average of 528 active patients per year, the prevalence rate of children with co-occurring ABI and ASD is 2.7%.

#### 3.1.2. Comorbidities and Impairments

One of the children had no neuropsychological data available, due to this data being in a different unit of the hospital and not being uploaded to medical records at the time of the review. Percentages were calculated based on dividing the number of children who were “impaired” in a domain by the number of children that were assessed in that neuropsychological domain.

The most neuropsychological impairments were in the domains of executive functioning (78%), processing speed (72.7%), attention (72.7%), literacy (71%), non-verbal IQ (61.5%), mathematical ability (50%), and IQ (50%). With regards to comorbidities, 92.9% of cases had at least one comorbid DSM-5 condition and 100% had at least one medical comorbidity. Comorbidities across the cases included the following: ADHD (64.3%); anxiety disorder (64.3%); hearing and/or vision impairments (64.3%); a speech and language disorder (57.1%), seizures (50%), ID (42.9%), ODD (21.4%), and sleep difficulties (28.6%).

#### 3.1.3. Age of ASD and ABI

**Figure 1:**
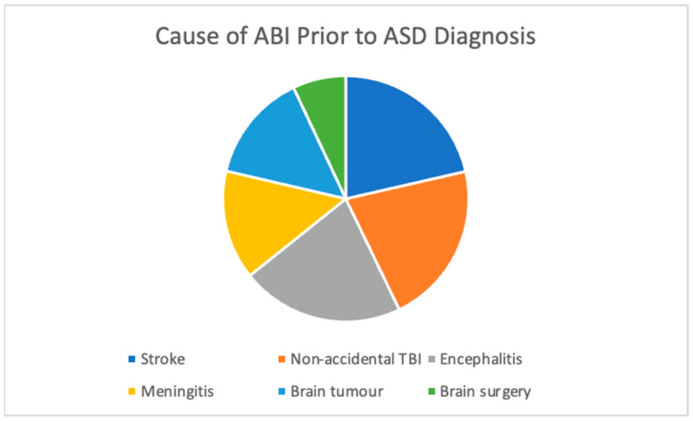
Cause of ABI in the Study Cohort for Patients with a Secondary ASD Diagnosis.

The mean age (Mage) at which the ABI was sustained for the cohort of 14 children with comorbid ASD was 18.66 months (or approximately 1.55 years, range: birth–118 months). Across all cases, the ABI occurred before an ASD diagnosis was given and symptoms occurred after and not prior to ABI. Figure 1 shows the nature of the ABI prior to a secondary diagnosis of ASD. Non-accidental TBI occurred in 21.4% of children while the remaining children had non-TBI related injuries (78.6%). Of the non-TBI related injuries, 21.4% were due to stroke, 21.4% were due to encephalitis, 14.3% were due to meningitis, and 14.3% were due to a brain tumour. One child had damage that occurred during brain surgery (7%).

Only one child had a family history of ASD (0.07%). The mean age of ASD diagnosis was 96.6 months or approximately 8 years and 5 months (range: 48–180 months), with the ADOS being the most common tool of diagnosis (35.7%). The average age of the first ASD symptom noted in medical records was 67.79 months or approximately five years and 6 months (range: 12 months to 150 months). In terms of ASD symptom onset, social difficulties were often the first difficulty to be reported by schools and/or parents (64.3%).

Six of the children (42.9%) were noted to have normal developmental milestones. Delayed language milestones were most common, presenting in seven children (50%). Five children had both motor and language milestones delayed (28.6%), with five children having only delayed motor milestones (35.7%).

Children who had 4+ comorbid DSM-5 diagnoses were diagnosed with ASD approximately two years later, on average (*n* = 6, Mage = 9.47), than those with 1 to 3 comorbid conditions (*n* = 7, Mage = 7.29). Children with two or more medical conditions (*n* = 7, Mage = 8.42) were diagnosed with ASD on average one year later than those with one medical comorbid condition (*n* = 7, Mage = 7.67). Children who had more than the average DSM-5 comorbid conditions (mean = 3.2, more than 2 conditions), were diagnosed with ASD approximately 1.5 years later (*n* = 10, Mage = 8.5) than those with the average number or less than the average number of comorbid DSM-5 conditions (*n* = 4, Mage = 6.9).

## 4. Discussion

### 4.1. Findings

#### 4.1.1. Prevalence of ASD following Paediatric ABI

The first aim of this study was to examine the estimated prevalence rates of children with comorbid ABI and ASD. In line with our prediction, the prevalence rate of ASD was higher in our paediatric ABI cohort than in the general population. In the general population, the estimated worldwide prevalence of ASD is somewhere between 0.60% and 1.85% [14]. Of note, the prevalence rate for ASD in the present study (2.7%) was higher than the prevalence rate of ASD reported by Chang et al. [9], that being 0.8%, in their study of children who sustained a TBI under the age of 3 years. One core difference between the present study and Chang et al.’s study was our incorporation of children who had sustained broad forms of ABI, extending beyond TBI. Another core difference was the average age at which the brain injury was sustained. The present study included ABIs sustained from birth to 9 years and 10 months, while Chang et al. only included children who sustained a TBI prior to the age of 3 years.

#### 4.1.2. Age of ABI as a Predictor of ASD

As for our second aim, in line with our predictions, and with the vulnerability hypothesis [36], younger age of ABI was seemingly important, as the mean age of ABI was 1.55 years for those diagnosed with secondary ASD. This may represent shared biological mechanisms in ABI and ASD, as well as disruption to normal brain development and re-wiring [10,36].

#### 4.1.3. Prevalence Rates of Comorbid Conditions

In line with our predictions and in line with findings from [15,16], children in the current study had a high prevalence of both comorbid medical and DSM-5 diagnoses, with 92.9% of the cohort having at least one comorbid DSM-5 diagnosis and 100% having at least one medical comorbidity.

Common neuropsychological deficits emerged for those with co-morbid ABI and ASD, with more than 50% of this cohort showing impaired attention, information processing speed, non-verbal IQ, and mathematic abilities. Almost 75% displayed executive functioning and literacy deficits. This is consistent with literature that suggests that common cognitive changes associated with both ABI and ASD include a decline in concentration, slowed speed of information processing, planning, and problem-solving abilities [56,57,58,59].

In the current cohort, the rates of comorbid conditions were higher (92.9%) than in those with ASD and no co-occurring ABI (72%; [60,61]) as well as in those with ABI and no co-occurring ASD (38% to 63%; Luis and Mittenberg, 2002). Of note, over 64% of the current sample had a DSM-5 diagnosis of ADHD, which is higher than previously reported rates of comorbidity (19% to 20%) in children with only ABI [62]. The rates of ADHD comorbidity in our current cohort are comparable to those found in ASD-only samples (40% to 70%) [59,63].

#### 4.1.4. Average Age of ABI and ASD Diagnosis

A third aim of the study was to evaluate the average age of ASD diagnosis in a cohort of children with co-occurring ABI, and to evaluate if comorbidities would be an important factor affecting the age of ASD diagnosis. The third hypothesis was supported, as the average age of ASD diagnosis in our ABI cohort was older and around five years later, on average, than the reported average age of diagnosis in a population of children with ASD and no co-occurring ABI [18]. Our reported average age of diagnosis was also beyond the age where signs and symptoms suggestive of ASD should be notable [64,65], at least in a population of children with ASD and no co-occurring ABI. Furthermore, our prevalence rates of ASD co-occurring with early ABI may be even higher than reported, as children discharged from the service may receive a later ASD diagnosis externally, which would not be recorded in the medical databases.

The mean age at ASD diagnosis was higher in the current study compared to the average reported in Chang et al. [9]’s study. These differences could be attributed to demographic differences, different family/genetic risk factors and backgrounds, or a limitation in our sample size in comparison to that of Chang et al. [9]. That said, comorbid neurodevelopmental conditions apart from ASD, ADHD, and DD in their cohort were not documented. It is, therefore, difficult to ascertain if our current cohort had more comorbid conditions as compared to Chang et al.’s cohort, and if this could have contributed to the differences in the average age of ASD diagnosis between the studies.

The present study found an indication that the number of comorbid conditions potentially played a role in delayed ASD diagnosis. Children who had four or more comorbid DSM-5 diagnoses were diagnosed with ASD over two years later than those with three or fewer comorbid conditions. Those with a number of DSM-5 comorbid conditions that were greater than or equal to the average number of comorbid conditions for this cohort were diagnosed with ASD approximately 1.5 years later than those with fewer than the average number of comorbid DSM-5 conditions. A possible explanation of the delayed age of ASD diagnosis could be due to ABI-related symptoms potentially “masking” early ASD symptomology given the overlap in symptoms between ABI and ASD [10]. Previous literature has found that emerging ASD symptomology may be automatically attributed to the previous ABI as opposed to ASD or may be obscured by the behavioural symptomology associated with ABI [10,15,16]. In line with this view, research indicates that clinicians are at risk of underdiagnosing a co-occurring disorder by mistakenly attributing the symptoms to the primary disorder or condition of ABI [66], especially given the shared neurological mechanisms at play [10]. Additionally, the children in this study not only had co-occurring ABI and ASD, but over 71.4% of the children had three or more comorbid DSM-5 diagnoses. The phenomenon of comorbidities “masking” ASD symptomology would also be in accordance with the current study finding that, on average, the age of first ASD symptoms noted in medical records was later than the average age suggested in the literature for the general ASD population [67,68].

### 4.2. Clinical Implications

The present study suggests that the prevalence of ASD in children with ABI is higher than in the general population. The study also found that the age of ASD diagnosis may be delayed in those with co-occurring ABI, and that comorbid conditions could be a factor in this delay. The findings related to the prevalence of comorbid ASD with ABI, as well as the delayed age of ASD diagnosis in these children, have important implications in relation to early intervention. Indeed, the mean age of ASD diagnosis in the present sample was beyond the age where intervention has been found to be most effective [31,65]. Additionally, this study’s finding that a high number of DSM-5 comorbidities [55] in children with ABI and co-occurring ASD continues to highlight the importance of managing and monitoring mental health and other emerging diagnoses. These results also emphasize the need to consider the possibility of ASD within the context of other co-morbidities early on, and to periodically monitor for developing ASD symptomology in children with early ABI.

### 4.3. Strengths and Limitations

The present study findings must be considered within the context of strengths, as well as limitations. Strengths of the present study included the novelty of the topic as well as the comprehensive and longitudinal approach looking at the profiles of these children. This study also had a number of limitations. Due to the nature of our design and small sample size, we were unable to make statements about the causality of our associations. Thus, we cannot ascertain if early ABI is the most prominent risk factor for a delayed ASD diagnosis, or if there are other factors to consider. The current study could also be impacted by sampling bias, due to participants coming from one geographical area that may influence the samples characteristics. Lastly, we examined one hospital, and more accurate rates of co-occurring ASD and ABI would be obtained through examination of all children’s hospitals in Australia.

### 4.4. Future Research

In order to evaluate causality in addition to more accurate prevalence rates of ASD and ABI and the risk of early ABI in developing ASD, a large longitudinal design needs to be employed that uses gold standard diagnostic assessment approaches. To maximise potential intervention, there is a need for investigation into the efficacy of early ASD screening in those with ABI from a young age.

Of note, two of the reported cases sustained their ABI due to abuse. Research suggests that experiencing paediatric abusive head trauma is associated with poorer functional outcomes that non-abusive head trauma [69,70], and children who experience abusive head trauma are at an even higher risk of developing neurological impairment [71,72]. Thus, more research in this area is needed as these children may be at an even higher risk of developing subsequent ASD.

This study also draws attention to issues arising from the limited use of “gold standard” ASD diagnostic tools, such as the ADOS and ADI-R [73]. One study conducted in Australia found that out of 105 practitioners who diagnosed ASD, only 47% administered the ADOS and 39% the ADI-R [73]. This issue could potentially lead to under diagnoses or misdiagnoses of ASD, as well as a misestimation of the current prevalence rates of ASD in Australia. This study, therefore, lends strength to arguments that current methods for diagnosing and investigating the prevalence of ASD in Australia need to become more rigorous and uniform in order to obtain accurate results [74].

### 4.5. Conclusions

The present research highlighted the potential importance of early paediatric ABI as a risk factor for ASD. The present study also showed evidence of a delayed ASD diagnosis following early ABI. Furthermore, this study highlighted the need for investigation into the efficacy of early ASD screening in children with primary ABI, to maximise opportunities for early detection and intervention. The current study’s findings suggest future merit in examining the prevalence rates of co-occurring ABI and ASD.

## Figures and Tables

**Table 1:** Demographic Information Summary **^1^**.

	Age of ABI (Months)	Cause of ABI	Age at ASD Dx (Months)	Birth	ATT Dx	MH Dx	Behaviour Problems	Speech and Language Disorder	ID	Hearing/ Vision Issues	Physical Issues	Sleep Issues	Seizure Epilepsy
1	1	Non-accidental	108	VD-T	ADHD	ANX; Self-Harm	ODD	RLD-Severe ELD-Mod	ID-Mild to Mod	Hypotonia	-	-	Y
2	28	Tumour	100	CS-T	ADHD	ANX; BD	ODD	SSD-mild	-	VI; Mild deafness	Ataxic Gait	-	Y
3	1	Non-accidental	66	VD-T	ADHD	ANX	ODD	Mild Stutter	-	AST	-	-	-
4	118	Encephalopathy	144	VD-T	-	GAD	-	RLD-Mild ELD-Severe	ID-Mild	-	-	-	-
5	At birth	Grade 4 haemorrhage	131	VD-PT	ADHD	ANX	-	-	-	Squint	-	-	-
6	2	ADEM	74	VD-T	ADHD	ANX	-	-	-	HL-mild	-	-	-
7	51	Stroke Tumour	93	CS-T	-	-	-	-	GDD	Nystagmus VI	-	-	Y
8	6	Herpes Encephalitis	108	CS-T	-	-	-	Mod delayed language skills	-	-	GMD-mild	Y	Y
9	3	Stroke	48	-	ADHD	ANX	-	RLD-Severe -	-	-	-	Y	-
10	36	Surgical	96	CS-T	-	ANX DEP	-	LD-severe	ID-Mod, GDD	-	-	-	Y
11	2	Meningitis	59	-	-	-	-	-	-	VI	-	-	Y
12	0.23 (1 week)	Brain Tumour	168	VD-T	ADHD	-	-	E/R LD-mild	ID-Mild	Nystagmus Hemianopia	R-Hemiplegia	-	Y
13	Birth	Hypoxia	86	CS-T	ADHD	ANX: Self-Harm	-	-	ID-mild/mod	-	-	Y	-
14	13	Encephalopathy	72	VD-PT	ADHD	-	-	-	-	Sensorineural HL	-	Y	-

Note: ADEM = acute disseminated encephalomyelitis, ADHD = attention deficit hyperactivity disorder, ANX = anxious symptomology, AST = astigmatism, ATT = attention, BD = body dysmorphia, CS = caesarean birth, DEP = depressive symptomology, Dx = diagnosis, ELD = expressive language delay, GAD = generalised anxiety disorder, GDD = global developmental delay, GMD = gross motor dysfunction, HL = hearing loss, ID = intellectual disability, IVF = IVF pregnancy, LD = language delay, MH = mental health, PT = premature delivery, RLD = receptive language delay, SLI = specific learning impairment, SSD = speech sound disorder, T = at-term delivery, VD = vaginal birth, VI = visual impairment, Y = yes, ^1^ Comments relating to the DSM-5 [55] ASD diagnostic criteria in Table 2 are further explained in the relevant case summary (See Appendix B). If this information is not highlighted, that indicates the information was not available to examiners.

**Table 2:** Neuropsychological Data Summary.

	Age at Assessment (Years)	FSIQ	VIQ	NVIQ	WM	PS	AT	VM	VisM	EF	Literacy	Math
Case 1	10.01	Impaired	Intact	Impaired	Impaired	Impaired	Intact	Impaired	-	Intact	-	Intact
Case 2	8.03	Intact	Intact	Intact	Intact	Impaired	Impaired	Intact	-	Impaired	-	-
Case 3	5.01	Intact	Intact	Intact	-	Impaired	Impaired	-	-	Impaired	-	-
Case 4	11.09	Impaired	Intact	Impaired	Impaired	Impaired	Impaired	Impaired	Intact	Impaired	-	-
Case 5	9.11	Intact	Intact	Intact	-	Intact	Impaired	Intact	-	Impaired	Impaired	Intact
Case 6	5.04	Intact	Intact	Intact	-	Impaired	Impaired	Intact	-	-	-	-
Case 7	7.09	Impaired ^1^	-	-	-	-	-	-	-	-	Intact	Intact
Case 8	4.05	-	Impaired	Impaired	-	-	-	-	-	-	-	-
Case 9	11	Intact	Impaired	Impaired	-	Impaired	Impaired	Impaired	Intact	Impaired	Impaired	Impaired
Case 10	16	Impaired	Impaired	Impaired	-	Impaired	Impaired	Impaired	-	-	Impaired	Impaired
Case 11	4.11	-	Impaired	Impaired	-	-	-	-	-	Impaired	-	-
Case 12	15.08	Impaired	Impaired	Impaired	Intact	Intact	Intact	Intact	-	Intact	Intact	Intact
Case 13	15:00	Impaired	Impaired	Impaired	Impaired	Impaired	Impaired	-	-	-	Impaired	Impaired
Case 14	15.02	Intact	Intact	Intact	Intact	Intact	Intact	Intact	Intact	Impaired	Impaired	Impaired

Note: FSIQ = Full-Scale Intellectual Quotient, VIQ = Verbal Intellectual Quotient, NVIQ = Nonverbal Intellectual Quotient, WM = working memory, PS = processing speed, AT = attention, VM = verbal memory, VisM = visual memory, EF = executive functioning. ^1^ The psychological raw scores and scaled scores for Case 7 were unable to be obtained due to being stored in the Child Development Unit (The Children Hospital at Westmead). Thus, scores for this case are not converted into *z*-scores. However, Case 7 was considered impaired on some domains based on the DSM-5 [55] diagnoses provided in medical. In addition, reports and reports by CDU psychologists that Case 7’s literacy and numerical skills were “around the typical level for a kindergarten student”. Percentile ranks less than or at the 5th were considered impaired. Attention and academic tasks were considered impaired due to having a DSM-5 [55] ADHD diagnosis and SLD-reading, written expression, and mathematics.

## Data Availability

Data is not made available for ethical reasons.

## Appendix A

**Table A1:** Neuropsychological results as z-scores; Case 7 has no neuropsychological data.

	Case 1	Case 2	Case 3	Case 4	Case 5	Case 6	Case 8	Case 9	Case 10	Case 11	Case 12	Case 13 ^4^	Case 14
**Age at Timepoint**	7.05/10.01	5.01/8.03	5.01	10.02/11.9	9.11	5.04	4.05	11.00	16.00	4.11	14.07/ 15.08	15.00	11.10/12.06/ 15.20
**Intellectual Functioning**													
**WPPSI-III/WISC-IV/V**													
Verbal Comprehension Index	−1.13/−0.47	0.40/0.00	0.67	**−2.73**/−1.60	−0.73	1.80		−0.93	**−3.67**		**−2.20/NE**	**Impaired** 4th%tile	−0.73/ −0.73/NE
Perceptual Reasoning Index	−1.07/**−1.67**										**−1.93/NE**	**Impaired** 1st%tile	0.00/0.53/NE
Fluid Reasoning Index		0.33/−0.4		−0.40/**−2.40**	−1.60	0.00		**−2.40**	**−3.27**				
Visual Spatial Index		NE/0.00	−1.00	**−2.06/−2.87**	−2.20			**−2.60**				**Impaired** 1st%tile	
Processing Speed Index	**−2.73/−1.80**	−1.20/**−1.87**	**−1.93**	−1.13/**−2.27**	−0.13	−1.40		**−3.67**	**−3.67**		−1.33/NE	**Impaired** 0.4st%tile	−1.00/ 0.00/NE
Working Memory Index	−1.53/**−2.33**	−1.00/−0.80		−1.00/−1.73							−1.33/NE	**Impaired** 0.1th%tile	1.53/ −1.33/NE
Full Scale IQ	**−1.93/−1.87**	−0.13/−0.73	−0.93	**−1.73/−2.20**	−1.40	0.47		**−2.47**	**−3.93**		**−2.07/NE**	**Impaired**	−0.80/ −0.73/NE
**DAS-II**													
Verbal Cluster							**−2.47**			**−1.67**			
Non-verbal Cluster							**−2.60**			−0.80			
Spatial										**−4.40**			
Global Cognitive Index										**−2.73**			
**Attention**													
**Tea-CH**													
Sky Search													
Number Correct		−0.33		1.00	−1.17						NE/−1.33		0.33
Time Per Target		−1.33		−1.00	**−2.33**						NE/0.00		−0.67
Attention Score		−1.00		−0.67	**−2.00**						NE/0.67		−0.67
Score!		**−2.33**		**−2.33**	−0.67			**−2.00/−2.67**	**−2.33**		NE/1.00		
Sky Search DT											NE/−0.67		−1.33
**CPT**													
Detectability	1.20					2.20							NE/1.40
Omissions	0.20					0.60							NE/−0.10
Commissions	1.30					3.00							NE/**3.00**
Conners-EC/3 Parent/Teacher													
Inattention/Hyperactivity			**2.60**			0.90							
Inattention		**3.20**				**3.90**		0.90/ **3.60**					
Hyperactivity/Impulsivity		**1.90**				0.70		0.90/ **3.60**					
ADHD_HI						0.30		1.2/ **3.20**					
ADHD_I						2.50		1.20/ **2.90**					
**Memory**													
**CMS**													
Stories Immediate	**−2.00**			**−2.33**		0.67					NE/−0.67		−1.00/ −0.67/0.66
Delayed	**−1.33**			**−2.67**		0.00					/NE−0.67		−0.33/−1.00/ −0.33
Recognition	−1.00				0.33					NE/−0.67	−0.67/**−1.67**/ −0.66		
Dots learning							**−3.00**/ −0.33				0.67		
Dots Immediate				−0.33				**−3.00** /0.00					1.00
Dots Delay				0.00				**−1.67** /NE					1.00
**CVLT-C/II**													
Trials 1–5 Correct	**−1.90**	0.60			0.50			**1.80** /NE	**−3.00**				NE/−0.60
Short Delay Free Recall	**−2.50**	0.50		0.00				**−2.00** /NE	**−3.00**				NE/0.00
Long Delay Free Recall	**−2.50**	0.00		0.50				−1.50 /NE	**−2.50**				NE/−0.50
**Executive Functions**													
**Tea-Ch**													
Creature Counting													
Total Correct				−1.00	**−2.33**						NE/0.33		
Timing Score				−0.67							NE/−1.00		
Walk, Don’t Walk		**−2.00**											
**NEPSY-II**													
Animal Sorting Combined		−1.33		**−2.67**	**−1.67**			**−2.67/** **−1.67**					NE/0.00
Word Generation Letter	−1.00	−0.66			−0.67			**NE** **/−2.00**		**−3.00**	NE/−0.67		NE/−0.33
Word Generation Semantic				−1.00	**−1.67**			**NE/** **−2.33**		**−2.33**	NE/−0.33		NE/−0.33
**BRIEF**													
Inhibit	1.13	0.60	**1.93**					**2.50**			NE/1.60		**1.80/2.20**
Shift	1.20	1.27	1.27					**2.40**			NE/**2.60**		**3.80/2.80** **/1.53**
Emotional Control	0.80	0.93	1.40					**3.20**			NE/0.00		**3.00/1.80**
Behaviour Regulation Index								**2.60**			NE/1.40		**3.33/2.60**
Initiate	1.40							**2.90**			NE/**2.90**		**1.5/1.53**
Working Memory	1.47	0.40	**2.53**					**3.50**			NE/**3.50**		**2.3/2.13**
Plan/Organise	1.53	0.73	**2.27**					**2.70**			NE/**2.90**		**2.0/1.80**
Organisation of Materials	0.89							**2.30**			NE/0.80		0.7/0.87
Monitor	0.73							**2.30**			NE/1.50		**4.70/1.67**
Metacognition Index/Cognitive Regulation Index	1.40	0.53	**2.60**					**3.10**			NE/**2.70**		**2.00**
Global Executive Composite	1.40	0.93	**2.47**								NE/**2.50**		**2.70**
**Academic Skills**													
**WIAT-II**													
Word Reading					**−1.87**			**−2.53/−2.80**	−1.07		NE/−1.07	**Impaired** 1st%tile	**−2.20/−2.53/** **−2.53**
Reading Comprehension					−1.5							**Impaired** 1st%tile	
Spelling					**−1.67**			**−2.13/−2.00**	**−2.40**			**Impaired** 1st%tile	**−2.26/−1.87/** **−2.40**
Numerical Operations	−0.93				−1.27			**−3.87**	**−4.00**		NE/−1.20	**Impaired** 1st%tile	**−1.40/** −1.26/ **−1.73**
Maths Problem Solving					**−1.73**			NE/**−2.67**				**Impaired** 1st%tile	
**Additional Behaviour Questionnaires**													
**Conners 3/EC**													
Learning Problems		**3.90**				0.40		**1.70/** **4.80**					
Executive Functioning		0.50				0.50		0.80/ **1.80**					
Defiance/Aggression	1.50	0.70				**2.10/** **4.00**		0.30/ 1.20					
Peer Relations		**2.20**				1.20		**2.80/** **0.90**					
Conduct Disorder								0.60/ 1.00					
Oppositional Defiant Disorder								0.10/ 1.40					
BASC−2/3 P/T													
Externalising Problems		**1.70**				0.70		/1.40	0.70				NE/NE/ **2.10**
Internalising Problems		**2.00**				1.60		/1.40	**2.50**				NE/NE /0.8
Behavioural Symptoms Index		**3.50**				1.00		/**1.80**	1.40				NE/NE /**2.40**
Activities of Daily Living		−2.90				0.10		/−1.50	−1.30				NE/NE/ −2.30

Note. All scores have been converted to z-scores. With the exception of the BRIEF, the Conners, CPT, and the three of the BASC scales (externalising problems, internalising problems, and behavioural symptoms index) 3, scores above −1.64 are considered intact; **at or below −1.64 are impaired (bolded)**. For the BRIEF, CPT, BASC scales (externalising problems, internalising problems, and behavioural symptoms index) and Conners 3, scores below 1.64 are considered intact; **at or above 1.64 are impaired (bolded)**. For ADIS-C data, only those disorders that met diagnostic criteria are included. NE = not examined. ^4^ The psychological raw scores and scaled scores for Case 13 were unable to be obtained due to being stored in the Child Development Unit (The Children Hospital at Westmead). Thus scores for this case are not converted into z-scores. However, Case 13 was considered impaired on some domains based on percentile ranks and DSM-V V (American Psychiatric Association, 2013) SLD diagnoses provided in medical reports. Percentile ranks less than or at the 5th were considered impaired. Attention was considered impaired due to having a DSM-5 V (American Psychiatric Association, 2013) ADHD diagnosis.

## Appendix B

- Case 1

Case 1 sustained a non-accidental, severe brain injury diagnosed at four weeks of age with an associated subdural hematoma noted on MRI. The history provided by Case 1’s parents was inconsistent with her injury. Case 1 was subsequently placed into protective care at eight months of age. A family history of learning difficulties (maternal) and Asperger’s syndrome (sibling, now referred to as ASD, level 1; DSM-5; American Psychiatric Association, 2013) was noted in medical records. No information about pregnancy or birth was available.

Case 1 was diagnosed with ASD using the ADOS (severity unknown) at nine years of age. ASD symptomology was noted from two years of age, with poor social skills being evident from this time. Developmental milestones were in the normal range at two years of age (commando crawling at nine months, walking at 12 months of age, saying 20+ words at two years of age). The overall level of development at 3 years and 9 months was placed in the 18- to 24-month range on formal assessment (CELF-preschool, Rossetti Infant Toddler Scale, Peabody Developmental Motor Scales), indicating severely delayed language, play, and social development. Subsequently, multidisciplinary intervention began. This included occupational therapy focusing on gross motor skills (continuing through to adolescence), physiotherapy (consistently until seven years of age) focusing on gross motor skill development, muscle strength, coordination and balance, and speech and language therapy to support phonological and language difficulties (until the age of 14 and then again at age 18). Case 1 also had a behavioural intervention to address social difficulties, sleeping, and eating compliance issues (continuing through adulthood). At three years of age, a 12-month history of hand flapping was noted, and a subsequent investigative sleep/wake EEG was normal.

Case 1 was in mainstream education with a modified curriculum and extensive teacher’s aide support in primary school and an ASD support unit for high school. At age seven, Case 1 was diagnosed with mild to moderate articulation difficulties, literacy difficulties, moderate to severely delayed receptive language and mildly delayed expressive language impairment. Difficulties noted during Case 1’s primary schooling included poor self-regulation and noise sensitivity (age five), repetitive motor mannerisms (age six), limited eye contact and echolalia (age 8), and immature social play and ritualistic behaviour (age 10 years 10 months). On repeat speech assessment in high school, Case 1 was noted to have severely delayed receptive language skills and moderately delayed expressive language skills, in addition to significant delays with reading decoding and comprehension.

- Case 2

Case 2 sustained a severe brain injury at 2 years and 4 months of age following an occipital craniotomy for a posterior fossa astrocytoma resection. MRI brain imaging (pre-surgery) indicated that the brainstem, cerebellar vermis, and fourth ventricle were compressed, and there was mild enlargement of the lateral and third ventricles and an atypical region in the inferior and medial areas of the right frontal lobe attributed to gliosis, myelination, or possible cortical dysplasia. The tumour was resected and resulted in significant post-surgery changes and impairments, including multiple ongoing cranial nerve palsies. An ASD diagnosis at age 8 years and 4 months was given by the treating paediatrician, reportedly utilising the diagnostic criteria in the DSM-5 (American Psychiatric Association, 2013). No notable family history was evident in medical reports.

Developmental milestones were typical (crawling at 7–8 months, walking at 14 months following one and two-step commands and producing four-word sentences at 4 years and 2 months). ASD symptomology was noted from the age of four and included rigid behaviour patterns, restrictive interests and coping poorly with change. Case 2 was supported by a special needs teacher in preschool. On a formalised questionnaire of behaviour completed by Case 2’s mother during preschool, difficulties were evident in controlling emotions and flexible thinking. A speech assessment at the age 5 and 10 months of age indicated average receptive and expressive language skills and difficulties with producing intelligible speech due to dysarthria.

Following this, multidisciplinary interventions began at the age of five. This included physiotherapy to improve balance (regularly at the age of five then ceased), occupational therapy to assist in developing fine motor control, speech pathology and behavioural intervention for emotional outbursts (all regularly documented up until the age of 10).

In primary school, Case 2 received additional support across a range of areas, including literacy. At age seven, Case 2 had difficulties switching between classroom activities. A speech assessment at this time identified average core language skills, a mild speech sound disorder, phonological difficulties and significant literacy problems impacting on all aspects of literacy, and social/pragmatic language problems. Subsequently, at 8 years and 3 months of age, Case 2 was noted to be annoyed by tags; insist on removing clothes if they had any dirt spots; have difficulties with social interactions and maintaining friends (e.g., having limited eye contact and difficulties with socials cues); have restricted interests, preoccupations with topics; and needing things to be in order. At one and a half years of age, in addition to the above behaviours, Case 2 was noted to exhibit hand flapping, bouncing behaviour, and making odd noises.

- Case 3

Case 3 sustained a severe non-accidental TBI at one month of age, resulting in a skull fracture, occipital contusion, right occipital horn intraventricular haemorrhage, and right posterior temporal subarachnoid haemorrhage. In addition, Case 3 was diagnosed with ASD (level 2 for social communication and level 3 for restricted repetitive behaviours) at 5 years and 6 months of age. Case 3 lived in out of home care with extended family. Notable family history includes maternal mental illness.

Developmental milestones were within the normal limits (crawling at 11 months, walking at 1 year 5 months, saying 30 words at 1 year 6 months). ASD symptomology was initially noted at 3 years and 5 months and included abnormal social interactions (e.g., avoiding group activities, parallel play) and communication (e.g., babyish talk). Following this, early multidisciplinary intervention for Case 3 began, including behavioural interventions involving parent–child interaction therapy (documented from the age of 3 until 7), occupational therapy (documented from preschool until the age of 7) and speech therapy (in preschool).

During preschool, at age 5 Case 3 was noted to be a picky eater and exhibit behavioural challenges associated with impulsivity, poor compliance, reduced danger awareness, high levels of physical activity, decreased attention span, and difficulty transitioning in activities. Case 3 was diagnosed with ASD following a speech assessment indicating poor conversational skills (e.g., lots of repetition, non-specific vocab interspersed with good vocab), and reports from school and caregivers of abnormal behaviours including phobias to loud noises, rigid and repetitive routines (e.g., repeating songs and avoiding certain textured foods), and poor social interaction (e.g., avoidance of group activities and limited eye contact). Case 3 was also noted to exhibit significant behavioural difficulties (e.g., defiant behaviour, anxious behaviour, emotional outbursts), which were managed with atypical antipsychotics and SSRIs.

Following notable episodes of violence that resulted in multiple suspensions during kindergarten, Case 3 was placed in a specialist support placement. At age seven, Case 3 continued to demonstrate defiant behaviour at school (e.g., damaging school property) and obsessive and restricted interests.

- Case 4

Case 4 was diagnosed with encephalopathy with associated seizures at the age of 9 years and 10 months following admission to hospital with seizures, an ataxic gait, an episode of incontinence and impaired short-term memory. ASD (Level 2) was diagnosed at 12 years of age by the Child Development Unit at the Children’s Hospital Westmead.

Developmental milestones were noted to be normal in medical reports. Case 4 was noted to have articulation difficulties before the ABI. However, Case 4 underwent speech intervention to assist with articulation difficulties (from the age of 10), occupational therapy (from the age of 10), physiotherapy (from the age of 10 until 11), and psychological intervention for anxiety management (from the age of 11). A speech pathology assessment conducted when Case 4 was 10 years and 2 months indicated that Case 4 had a severe language disorder characterised by moderate receptive language impairment, severe expressive language impairment, and severe higher-level language impairment.

Case 4 attended a mainstream school until 11 years of age, after which she was home-schooled until the age of 12. Concerns regarding social interaction with peer groups began when Case 4 was 10 years of age and included Case 4 often sitting alone at recess and not having any reported friends at school. Case 4 developed anxiety at the age of 11, particularly around exposure to germs and loud noises. At the age of 11 Case 4 was had poor communication skills (with her speech often including babbling, confusing real with make-believe, difficulties understanding sarcasm or humour), have concrete reasoning and fixed routines and rituals, and unusual sensitivities (e.g., asking to smell others’ hands).

- Case 5

Case 5 had a grade 4 intraventricular haemorrhage on the left side complicated by obstructive hydrocephalus following a twin-to-twin transfusion at birth. She subsequently had a Rickman’s reservoir inserted at 3 weeks of age and a left VP shunt insertion at the age of 2½ months. Case 5 was diagnosed with ASD at 10 years and 11 months of age via the ADOS.

Case 5 was born prematurely at 31 weeks gestation. Following the intraventricular haemorrhage at birth, she spent six weeks in the NICU. Case 5’s developmental motor milestones were delayed (rolling at 10–12 months, crawling at one year) but her language skills developed in the normal ranges (25+ words at two years). Early intervention included physiotherapy and occupational therapy fortnightly (eight months to current), speech therapy (regularly from two years until unknown age), and psychology therapy for anxiety (irregularly from age nine to current).

Case 5 attended mainstream schooling. An ASD screener (ASRS) was given to Case 5’s mother and teacher when Case 5 was nine years of age. This screener indicated elevated symptoms associated with ASD across all settings, particularly in unusual behaviours, rigidity, self-regulation, and attention. Cases 5’s mother also reported ongoing learning difficulties and social challenges, including having difficulty maintaining friendships, not understanding social cues and nuances, being rigid in thinking, and struggling to deal with changes to routine.

- Case 6

Case 6 suffered an ABI following two bouts of acute disseminating encephalomyelitis (ADEM), first occurring at the age of 4 years and 3 months of age then suffered a relapse 6 weeks later. An ASD diagnosis was given to Case 6 at the age of 6 years and 2 months of age via the ADOS.

Case 6 met all developmental milestones on time. In preschool, concerns were noted around his ability to follow instructions as well as his social and emotional skills. Early intervention included speech therapy (at the age of five until seven) and occupational therapy focusing on handwriting and emotional regulation skills (at the age of five until current).

Case 6 commenced kindergarten in a mainstream school with a teacher’s aide. Concerns noted by Case 6’s teacher included the following: increased difficulties concentrating; poor comprehension of multiple-step instructions; emotional/behavioural outbursts; inability to follow social cues; difficulties with interacting with peers and age-appropriate play. Case 6’s parents noted that Case 6 could get stuck on topics and had a strong preference to play on an iPad.

- Case 7

At seven weeks of age, Case 7 was identified to have a neonatal tumour. Biopsy surgery at eight weeks of age was complicated by a stroke involving the left middle cerebral artery and posterior cerebral artery infarctions. At 7 years and 9 months, the ADOS was used to diagnose Case 7 with ASD (level 3 for social communication and repetitive restrictive behaviour). There is no relevant family history other than an unborn sibling who was antenatally diagnosed with Trisomy 13.

Following Case 7’s stroke, regular (fortnightly or weekly) early intervention included physiotherapy, occupational therapy, and speech therapy, from one year of age until currently. Psychological therapy was conducted for behavioural management from four years of age until currently. Case 7 participated in weekly hydrotherapy from the age of two until currently. Developmental motor and language milestones were delayed (sitting at two years, first words at four years of age).

Case 7 attended a mainstream school in a support unit for children with a moderate intellectual disability. At seven years of age, Case 7 was noted to be fussy with food, have fixed ideas and interests (e.g., trains), difficulties with social niceties and social behaviour, difficulties with changes in routine, and not liking loud noises. Case 7 also demonstrated abnormal behaviours such as flapping hands and rocking when excited, needing things arranged a certain way, and mouthing and chewing toys. Following these concerns, an ADOS assessment showed that Case 7 presented with symptoms of ASD, including limited social reciprocity, limited variation in facial expression, basic imaginary play, and repetitive language.

- Case 8

Case 8 had herpes simplex encephalitis (Type 1), at six months of age. The encephalitis caused specific areas of brain injury, which includes her right temporal and parietal lobes as well as small areas of damage in the left parietal, left temporal, and right frontal lobes. ASD was diagnosed at 3 years and 5 months of age following a developmental assessment. Case 8’s language development (at 11 months, vocabulary consisted of about five words and increased to 15–20 words by 2 years 5 months) and motor development were within normal limits (sitting unsupported by 11 months, walking alone at 13 months).

Early interventions included regular occupational, speech, and language therapy and physiotherapy intervention from infancy until she was five years of age. At about two years of age, her neurologist noted she was not socially interactive, took little notice of what was happening around her, and seemed to be in her own world and unengaged. At the age of 3 years 5 months, a developmental assessment (Bayley’s Scale of Infant Development 3rd Edition) identified that Case 8 had moderately delayed language skills. In terms of her social functioning and behaviour, Case 8 was a child who liked to play alone without social reference or attempts to share her enjoyment, preferred to direct her play, was distractible, and often wandered in her environment. At the age of 4 years and 5 months, Case 8 had rigid routines (e.g., having colours in a certain order and lining up toys), inflexible thinking, difficulties sleeping, and sensory issues. Sensory issues included touching her food before she eats it and hating having her hair brushed. Case 8 was placed in a small class with teaching support in a mainstream school for her primary schooling years.

- Case 9

Case 9 was born with complex cyanotic congenital heart disease, for which he had two major open-heart operations. A left frontoparietal cerebral vascular infarct (three months of age) and a global watershed-type infarct (four years of age) complicated these operations. A subsequent ASD diagnosis by Case 9’s paediatrician was made at four years of age.

Following his ABI, Case 9’s early intervention included the following: regular (weekly) speech therapy; occupational therapy; behavioural intervention; physiotherapy on and off until the age of 17. Case 9’s developmental milestones were delayed (communication and motor difficulties).

Case 9 attended a mainstream school in primary school and repeated year one. At five years of age, Case 9 was noted to be inattentive, impulsive, distractible, a fussy eater, perseverative, and had a poor understanding of social boundaries. Additionally, Case 9 had fixed interests, difficulty sharing, and poor language skills. A speech pathology assessment at six years of age revealed severe delays in his receptive language skills. Case 9 attended a multicategory support class in a mainstream school with a teacher’s aide to assist him in high school.

- Case 10

Case 10 underwent left frontal lobe resection epilepsy surgery at three years of age. A diagnosis of ASD by his paediatrician occurred at eight years of age.

Case 10 had normal motor development but was delayed in his language and social milestones (did not speak much at all up until age 3–4, vocabulary consisted of 20 words by age four). At age two, Case 10 was assessed using the Griffiths Mental Development Scales and was diagnosed with a moderate developmental disability. At the age of 3 years and 2 months, speech pathology assessment documented a severe language delay. Further, at the time of this assessment, Case 10 was noted to make fleeting eye contact, have poor comprehension, and social difficulties (e.g., lashing out at others, enjoying solitary play compared to group play). Subsequently, Case 10 attended speech therapy from the age of three until four, and he also attended occupational therapy in his teens infrequently.

Case 10 attended a learning support unit in a mainstream school with the support of a teacher’s aide. In high school, Case 10 was distractible in the classroom. Case 10 was assessed by a psychiatrist at the age of 7 due to severe behavioural difficulties, including hyperactivity, self-harm, aggression, and repetitive behaviours.

At the age of 14 years and 6 months of age, Case 10 was noted to have restricted interests and was taken advantage of by other students in the classroom. At the age of 16, a formalised measure of behaviour (the BASC-3) completed by Case 10’s parents indicated that he had clinically significant elevated levels of anxiety, depression, internalising problems, and atypicality.

- Case 11

Case 11 suffered a brain injury from Haemophilus influenzae type B meningitis at 2 months of age. This resulted in extensive cortical necrosis involving the right frontal, left parietal, left temporal, and bilateral occipital lobes. Case 11 was diagnosed with ASD at the age of 4 years and 11 months following a neuropsychological assessment.

Case 11 was admitted to the hospital with symptoms of meningitis at two months of age. He was initially admitted to the Paediatric Intensive Care Unit for 12 days and then to the ward for another 12 days. During his stay, Case 11 received multidisciplinary review and assessment including physiotherapy, speech pathology and occupational therapy until his discharge. Subsequently, multidisciplinary therapy included ABA therapy to assist with language and behavioural skills (from preschool years), and occupational therapy, speech therapy, and physiotherapy on an as-required basis. Developmental milestones were delayed for language (two-word sentences at three years of age) and motor abilities (walking only 2–4 m at the age of 2 years and 5 months).

At two years of age, Case 11 was noted to be a fussy eater and have an interest in spinning objects. Additionally, at 2 years and 9 months of age, Case 11 had difficulties with following routines within the room and struggling with transition in routine in the classroom (preschool). A school observation was also conducted at this time by an occupational therapist in which Case 11 was noted to be irritated by the proximity of others, require significant encouragement to sit down, engage in some self-stimulation behaviours in play (e.g., spinning, flapping), and to resort to banging his head, kicking, and scratching when he was unable to cope with the transitions.

At 3 years and 5 months of age, Case 11 was noted to engage in parallel play and often copy other children but did sometimes take turns. Case 11 attended preschool with the assistance of an ABA therapist. At the age of 4 years and 11 months, Case 11 was noted to be aggressive if he did not get his way, have obsessive and restrictive interests, and difficulties initiating play. During a neuropsychological assessment, Case 11 was also noted to have marked social impairment (e.g., lack of social or emotional reciprocity and impaired eye contact), impairments in communication (e.g., delayed spoken language skills, repetitive use of language, and lack of make-believe play), and restricted, repetitive, and stereotyped patterns of behaviour (e.g., flapping hands when distressed and restricted patterns of interest). At the age of five, Case 11 was noted to have some communication improvements and demonstrated less self-stimulatory behaviour but still spun if tense or if his routine has been disturbed without preparation. In primary school, Case 11 attended a support class in a mainstream school. Social skills continued to be a difficulty for Case 11 in primary school and he was noted to have significant behavioural outbursts.

- Case 12

Case 12 had a brain tumour (left temporoparietal ganglioglioma) present from birth. As a result, he required neurosurgery at 7 days of age. This tumour resulted in extensive cystic changes within the left cerebral hemisphere and extensive damage to the left frontal, left parietal, and left temporal lobes. He was diagnosed with ASD before the age of 14 years old. However, records do not indicate an exact age or method of diagnosis.

Case 12 was removed from one of his parents at a young age due to abuse, thus milestone and birth information was not available. As a result of the surgery and tumour, Case 12 experienced physical and developmental problems, as well as language and cognitive problems. Following this, early multidisciplinary intervention included the following: occupational therapy for fine and gross motor skills, physiotherapy for muscle tone; speech therapy; psychology therapy addressing problems with communication.

Case 12 initially attended a mainstream school for his primary school years. Case 12 attended an early intervention programme for visually impaired children from 6 months of age. At the age of eight, Case 12 had significant behaviour problems, was prone to angry outbursts, found it challenging to follow conversations, and was noted to have obsessive behaviours and rigidity. Case 12 was placed in a mainstream class briefly for Year 9 and then transferred to a multi-category supported class (within a mainstream high school) on the basis of an Asperger’s Syndrome diagnosis (now referred to as ASD; DSM-5; American Psychiatric Association, 2013) and intellectual impairment for Year 10.

- Case 13

Case 13 required resuscitation at birth and oxygen for 36 h and was diagnosed with Phenylketonuria (PKU) at birth. A paediatrician made a subsequent ASD diagnosis at the age of 7 years and 2 months based on developmental history.

Language milestones were delayed (speaking 25 words at three years) but motor milestones were normal (walking at two years of age, sitting at eight months, crawling at 15 months).

Case 13 had psychological intervention (for anxiety, anger, and thoughts of self-harm), physiotherapy and occupational therapy on and off from 12 months of age, and speech therapy on and off from two years of age until currently. At the age of 4 years and 5 months, a speech pathology assessment indicated that Case 13’s receptive language skills were in the low average range whilst expressive language was in the average range.

Case 13 attended a multi-category support class at school. At the age of 7 years and 2 months, Case 13 displayed an increased amount of stereotyped behaviour such as flapping her hands and a tendency to twiddle with her hair and push holes in her socks. Case 13 was subsequently diagnosed with ASD based on developmental history and observations of her play behaviour, adherence to routine, sensory sensitivities, and repetitive abnormal behaviours (arms flapping). At the age of 15, a comprehensive interview regarding Case 13’s adaptive skills (Vineland Adaptive Behaviour Scales-3) indicated her adaptive skills were well below the level expected, with particular difficulties with daily living skills and socialisation. Case 13’s carers responses on a developmental behavioural checklist at age 15 indicated concerns related to disruptive behaviour (e.g., emotional outbursts, stubborn tendencies, impulsive tendencies), low mood (e.g., feeling down, withdrawn, lack of enjoyment), and anxiety (e.g., over-excited, resists change). Ongoing concerns with relating to her peers and communicating with others were also reported.

- Case 14

Case 14 had a possible encephalopathy at 13 months of age, following receiving the measles-mumps-rubella vaccination. He developed a high fever, progressing to a rash and had clear evidence of global developmental regression and sensory neural hearing loss at this time. A PET scan demonstrated relative mild patchy reductions in cerebral metabolism in the anterior parietal lobes bilaterally, generalised reduced tracer uptake within the temporal lobes and cerebellum, and some evidence of CSF inflammation. At the age of 6 years, Case 14 was formally diagnosed with ASD using the ADOS by his paediatrician.

Initial motor and language milestones were normal (walking at 12 months, speaking 15 words at 12 months). Case 14 went to a mainstream school with teacher’s aide support. At the age of 11, Case 14 was admitted to hospital for a thorough medical investigation and further neuropsychological assessment due to ongoing concern around the deterioration of his cognitive abilities over the course of 18 months to two years. At 12 years and 6 months of age, Case 14 underwent a speech and language review that indicated receptive and expressive language skills were within normal limits. However, difficulties were noted with his ability to retain and recall spoken information and his social skills (e.g., understanding other’s feelings). At age 15 and 2 months, Case 14’s carers indicated that his overall level of adaptive functioning was below average. His conceptual, social, and practical skills were all rated generally in the low range for his age.

## References

[1] Muhle R., Trentacoste S.V., Rapin I. (2004). The genetics of autism. Pediatrics.

[2] Singletary W.M. (2015). An integrative model of autism spectrum disorder: ASD as a neurobiological disorder of experienced environmental deprivation, early life stress and allostatic overload. Neuropsychoanalysis.

[3] Yoo H. (2015). Genetics of autism spectrum disorder: Current status and possible clinical applications. Exp. Neurobiol..

[4] Li X., Zou H., Brown W.T. (2012). Genes associated with autism spectrum disorder. Brain Res. Bull..

[5] Xiang A.H., Wang X., Martinez M.P., Walthall J.C., Curry E.S., Page K., Buchanan T.A., Coleman K.J., Getahun D. (2015). Association of Maternal Diabetes with Autism in Offspring. JAMA J. Am. Med. Assoc..

[6] Cohen M. (1997). Children’s Memory Scale (CMS).

[7] Wu T.C., Wilde E.A., Bigler E.D., Li X., Merkley T.L., Yallampalli R., McCauley S.R., Schnelle K.P., Vasquez A.C., Chu Z. (2011). Longitudinal Changes in the Corpus Callosum following Pediatric Traumatic Brain Injury. Dev. Neurosci..

[8] Modabbernia A., Velthorst E., Reichenberg A. (2017). Environmental risk factors for autism: An evidence-based review of systematic reviews and meta-analyses. Mol. Autism.

[9] Chang H.K., Hsu J.W., Wu J.C., Huang K.L., Chang H.C., Bai Y.M., Chen T.J., Chen M.H. (2018). Traumatic brain injury in early childhood and risk of attention-deficit/hyperactivity disorder and autism spectrum disorder: A nationwide longitudinal study. J. Clin. Psychiatry.

[10] Singh R., Turner R.C., Nguyen L., Motwani K., Swatek M., Lucke-Wold B.P. (2016). Pediatric Traumatic Brain Injury and Autism: Elucidating Shared Mechanisms. Behav. Neurol..

[11] Ewing-Cobbs L., Prasad M.R., Swank P., Kramer L., Cox C.S., Fletcher J.M., Barnes M., Zhang X., Hasan K.M. (2008). Arrested development and disrupted callosal microstructure following pediatric traumatic brain injury: Relation to neurobehavioral outcomes. Neuroimage.

[12] Cozolino L., Sprokay S. (2006). Neuroscience and adult learning. New Dir. Adult Contin. Educ..

[13] Ryan N.P., Catroppa C., Godfrey C., Noble-Haeusslein L.J., Shultz S.R., O’Brien T.J., Anderson V., Semple B.D. (2016). Social dysfunction after pediatric traumatic brain injury: A translational perspective. Neurosci. Biobehav. Rev..

[14] Lord C., Brugha T.S., Charman T., Cusack J., Dumas G., Frazier T., Jones E.J.H., Jones R.M., Pickles A., State M.W. (2020). Autism spectrum disorder. Nat. Rev. Dis. Prim..

[15] Levy S.E., Giarelli E., Lee L.C., Schieve L.A., Kirby R.S., Cunniff C., Nicholas J., Raven J., Rice C.E. (2010). Autism spectrum disorder and co-occurring developmental, psychiatric, and medical conditions among children in multiple populations of the United States. J. Dev. Behav. Pediatr..

[16] Stevens T., Peng L., Barnard-Brak L. (2016). The comorbidity of ADHD in children diagnosed with autism spectrum disorder. Res. Autism Spectr. Disord..

[17] Hyman S.L., Levy S.E., Myers S.M. (2020). Identification, evaluation, and management of children with autism spectrum disorder. Pediatrics.

[18] Van ’t Hof M., Tisseur C., van Berckelear-Onnes I., van Nieuwenhuyzen A., Daniels A.M., Deen M., Hoek H.W., Ester W.A. (2021). Age at autism spectrum disorder diagnosis: A systematic review and meta-analysis from 2012 to 2019. Autism.

[19] Miodovnik A., Harstad E., Sideridis G., Huntington N. (2015). Timing of the diagnosis of attention-deficit/hyperactivity disorder and autism spectrum disorder. Pediatrics.

[20] Falkmer T., Anderson K., Falkmer M., Horlin C. (2013). Diagnostic procedures in autism spectrum disorders: A systematic literature review. Eur. Child Adolesc. Psychiatry.

[21] Satterstrom F.K., Walters R.K., Singh T., Wigdor E.M., Lescai F., Demontis D., Kosmicki J.A., Grove J., Stevens C., Bybjerg-Grauholm J. (2019). Autism spectrum disorder and attention deficit hyperactivity disorder have a similar burden of rare protein-truncating variants. Nat. Neurosci..

[22] Courchesne E., Pierce K., Schumann C.M., Redcay E., Buckwalter J.A., Kennedy D.P., Morgan J. (2007). Mapping early brain development in autism. Neuron.

[23] Tick B., Bolton P., Happé F., Rutter M., Rijsdijk F. (2016). Heritability of autism spectrum disorders: A meta-analysis of twin studies. J. Child Psychol. Psychiatry.

[24] Deng W., Zou X., Deng H., Li J., Tang C., Wang X., Guo X. (2015). The relationship among genetic heritability, environmental effects, and autism spectrum disorders: 37 pairs of ascertained twin study. J. Child Neurol..

[25] Gaugler T., Klei L., Sanders S.J., Bodea C.A., Goldberg A.P., Lee A.B., Mahajan M., Manaa D., Pawitan Y., Reichert J. (2014). Most genetic risk for autism resides with common variation. Nat. Genet..

[26] Dennis M., Yeates K.O., Taylor H.G., Fletcher J.M., Stern Y. (2007). Brain reserve capacity, cognitive reserve capacity, and age-based functional plasticity after congenital and acquired brain injury in children. Cognitive Reserve: Theory and Applications.

[27] Baron-Cohen S., Auyeung B., Nørgaard-Pedersen B., Hougaard D.M., Abdallah M.W., Melgaard L., Cohen A.S., Chakrabarti B., Ruta L., Lombardo M.V. (2015). Elevated fetal steroidogenic activity in autism. Mol. Psychiatry.

[28] Frustaci A., Neri M., Cesario A., Adams J.B., Domenici E., Dalla Bernardina B., Bonassi S. (2012). Oxidative stress-related biomarkers in autism: Systematic review and meta-analyses. Free Radic. Biol. Med..

[29] Getahun D., Fassett M.J., Peltier M.R., Wing D.A., Xiang A.H., Chiu V., Jacobsen S.J. (2017). Association of perinatal risk factors with autism spectrum disorder. Am. J. Perinatol..

[30] Roberts A.L., Lyall K., Rich-Edwards J.W., Ascherio A., Weisskopf M.G. (2013). Association of maternal exposure to childhood abuse with elevated risk for autism in offspring. JAMA Psychiatry.

[31] Dawson G., Jones E.J.H., Merkle K., Venema K., Lowy R., Faja S., Kamara D., Murias M., Greenson J., Winter J. (2012). Early behavioral intervention is associated with normalized brain activity in young children with autism. J. Am. Acad. Child Adolesc. Psychiatry.

[32] Grossmann T., Johnson M.H. (2007). The development of the social brain in human infancy. Eur. J. Neurosci..

[33] Kuzniewicz M.W., Wi S., Qian Y., Walsh E.M., Armstrong M.A., Croen L.A. (2014). Prevalence and neonatal factors associated with autism spectrum disorders in preterm infants. J. Paediatr..

[34] Anderson V., Northam E., Wrennall J. (2018). Developmental Neuropsychology: A Clinical Approach.

[35] Friedland D., Hutchinson P. (2013). Classification of traumatic brain injury. Adv. Clin. Neurosci. Rehabil..

[36] Sariaslan A., Sharp D.J., D’Onofrio B.M., Larsson H., Fazel S. (2016). Long-Term Outcomes Associated with Traumatic Brain Injury in Childhood and Adolescence: A Nationwide Swedish Cohort Study of a Wide Range of Medical and Social Outcomes. PLoS Med..

[37] Wechsler D. (2003). Wechsler Intelligence Scale for Children.

[38] Wechsler D. (2014). Technical and Interpretive Manual for the Wechsler Intelligence Scale for Children.

[39] Wechsler D. (2012). Wechsler Preschool and Primary Scale of Intelligence.

[40] Elliott C.D., Murray G.J., Pearson L.S. (1990). Differential Ability Scales.

[41] Wechsler D. (2001). Wechsler Individual Achievement Test.

[42] Conners C.K., Staff M.H.S., Connelly V., Campbell S., MacLean M., Barnes J. (2000). Conners’ continuous performance Test II (CPT II v. 5). Multi-Health Syst. Inc..

[43] Manly T., Robertson I.H., Anderson V., Nimmo-Smith I. (1999). The Test of Everyday Attention (TEA-CH).

[44] Conners C.K. (2001). Conners’ Rating Scales-Revised: CRS-R.

[45] Conners C.K., Pitkanen J., Rzepa S.R., Kreutzer J.S., DeLuca J., Caplan B. (2011). Conners 3rd Edition (Conners 3. Conners 2008). Encyclopedia of Clinical Neuropsychology.

[46] Conners C.K., Goldstein S. (2009). Conners Early Childhood: Manual.

[47] Delis D.C., Kramer J.H., Kaplan E., Ober B.A. (2000). California Verbal Learning Test.

[48] Delis D.C. (1994). California Verbal Learning Test.

[49] Brooks B.L., Sherman E.M., Strauss E. (2009). NEPSY-II: A developmental neuropsychological assessment. Child Neuropsychol..

[50] Gioia G.A., Isquith P.K., Guy S.C., Kenworthy L. (2000). Behavior Rating of Executive Function.

[51] Kamphaus R.W., Reynolds C.R. (2007). Behavior Assessment System for Children.

[52] Kamphaus R.W., Reynolds C.R. (2015). Behavior Assessment System for Children.

[53] Pawela C., Brunsdon R.K., Williams T.A., Porter M., Dale R.C., Mohammad S.S. (2017). The neuropsychological profile of children with basal ganglia encephalitis: A case series. Dev. Med. Child Neurol..

[54] Williams T.A., Brunsdon R.K., Burton K.L., Drevensek S., Brady C., Dale R.C., Mohammad S.S. (2019). Neuropsychological outcomes of childhood acute necrotizing encephalopathy. Brain Dev..

[55] American Psychiatric Association (2013). Diagnostic and Statistical Manual of Mental Disorders.

[56] Leung R.C., Vogan V.M., Powell T.L., Anagnostou E., Taylor M.J. (2016). The role of executive functions in social impairment in Autism Spectrum Disorder. Child Neuropsychol..

[57] Mayes S.D., Calhoun S.L. (2003). Ability Profiles in Children with Autism: Influence of Age and IQ. Autism.

[58] Pellicano E. (2007). Links between theory of mind and executive function in young children with autism: Clues to developmental primacy. Dev. Psychol..

[59] Ivanović I. (2021). Psychiatric Comorbidities in Children With ASD: Autism Centre Experience. Front. Psychiatry.

[60] Leyfer O.T., Folstein S.E., Bacalman S., Davis N.O., Dinh E., Morgan J., Tager-Flusberg H., Lainhart J.E. (2003). Comorbid psychiatric disorders in children with autism: Interview development and rates of disorders. J. Autism Dev. Disord..

[61] Luis C.A., Mittenberg W. (2002). Mood and Anxiety Disorders Following Pediatric Traumatic Brain Injury: A Prospective Study. J. Clin. Exp. Neuropsychol..

[62] Max J.E., Lansing A.E., Koele S.L., Castillo C.S., Bokura H., Schachar R., Collings N., Williams K.E. (2004). Attention deficit hyperactivity disorder in children and adolescents following traumatic brain injury. Dev. Neuropsychol..

[63] Joshi G., Faraone S.V., Wozniak J., Tarko L., Fried R., Galdo M., Furtak S.L., Biederman J. (2017). Symptom Profile of ADHD in Youth with High-Functioning Autism Spectrum Disorder: A Comparative Study in Psychiatrically Referred Populations. J. Atten. Disord..

[64] Dawson G. (2008). Early behavioral intervention, brain plasticity, and the prevention of autism spectrum disorder. Dev. Psychopathol..

[65] Vismara L.A., Rogers S.J. (2010). Behavioral treatments in autism spectrum disorder: What do we know?. Annu. Rev. Clin. Psychol..

[66] Luiselli J.K. (2014). Children and Youth with Autism Spectrum Disorder (ASD): Recent Advances and Innovations in Assessment, Education, and Intervention.

[67] Baghdadli A., Pascal C., Grisi S., Aussilloux C. (2003). Risk factors for self-injurious behaviours among 222 young children with autistic disorders. J. Intellect. Disabil. Res..

[68] Chawarska K., Campbell D., Chen L., Shic F., Klin A., Chang J. (2011). Early generalized overgrowth in boys with autism. Arch. Gen. Psychiatry.

[69] Adamo M.A., Drazin D., Smith C., Waldman J.B. (2009). Comparison of accidental and nonaccidental traumatic brain injuries in infants and toddlers: Demographics, neurosurgical interventions, and outcomes. J. Neurosurg. Pediatr..

[70] Keenan H.T., Runyan D.K., Nocera M. (2006). Child outcomes and family characteristics 1 year after severe inflicted or noninflicted traumatic brain injury. Pediatrics.

[71] Lind K., Toure H., Brugel D., Meyer P., Laurent-Vannier A., Chevignard M. (2016). Extended follow-up of neurological, cognitive, behavioral and academic outcomes after severe abusive head trauma. Child Abuse Negl..

[72] Nuño M., Ugiliweneza B., Zepeda V., Anderson J.E., Coulter K., Magana J.N., Drazin D., Boakye M. (2018). Long-term impact of abusive head trauma in young children. Child Abuse Negl..

[73] Whitehouse A.J.O., Evans K., Eapen V., Wray J. (2018). A National Guideline for the Assessment and Diagnosis of Autism Spectrum Disorders in Australia.

[74] Williams K., Helmer M., Duncan G.W., Peat J.K., Mellis C.M. (2008). Perinatal and maternal risk factors for autism spectrum disorders in New South Wales, Australia. Child Care Health Dev..

